# *Calystegia soldanella* Extract Exerts Anti-Oxidative and Anti-Inflammatory Effects via the Regulation of the NF-κB/Nrf-2 Pathways in Mouse Macrophages

**DOI:** 10.3390/antiox10101639

**Published:** 2021-10-18

**Authors:** Taekil Eom, In-Hye Kim, Hyung-Joo Kim, YounHee Choi, Taek-Jeong Nam

**Affiliations:** 1 Institute of Fisheries Science, Pukyong National University, Pusan 46041, Korea; taekil7@pknu.ac.kr (T.E.); mercyih@naver.com (I.-H.K.); kimhj940@nver.com (H.-J.K.); 2 Department of Marine Bio-Materials & Aquaculture, College of Fisheries Science, Pusan 48513, Korea

**Keywords:** *Calystegia soldanella*, halophyte, inflammation, NF-κ, Nrf-2

## Abstract

Plant polyphenols are widely used to treat various inflammatory diseases, owing to their ability to suppress reactive oxygen species production and the expression of inflammatory cytokines. Herein, we investigated phenolic compounds from *Calystegia soldanella* using UPLC Q-TOF MS/MS and their antioxidative and anti-inflammatory activities were analyzed. The *C*. *soldanella* ethyl acetate fraction (CsEF) had the strongest antioxidative activity, given its high polyphenol compound content. It also exhibited anti-inflammatory effects, inhibiting the production of inflammatory cytokines such as NO, PGE2, IL-1β, IL-6, and TNF-α in LPS-stimulated mouse macrophages. CsEF activated the nuclear transcription factor Nrf-2, thereby upregulating antioxidant enzymes such as HO-1 and NQO-1 and inhibiting NF-κB expression, which in turn, suppressed the expression of COX-2, iNOS, and inflammatory cytokines, ultimately exerting anti-inflammatory effects. Further, UPLC-Q-TOF-MS/MS was used to analyze the polyphenol compound contents in CsEF. The quercetin glycosides isoquercitrin and quercitrin were the primary flavonoid compounds, while the caffeic acid derivatives, chlorogenic acid and dicaffeoylquinic acid, were the primary phenolic acids. Thus, *C. soldanella*, which had only a limited use thus far as a medicinal plant, may serve as a natural medicinal resource for treating inflammatory diseases.

## 1. Introduction

Reactive oxygen species (ROS), which are generated when aerobic organisms use oxygen to produce energy, play various roles in cells, including intracellular signaling and the regulation of homeostasis. However, ROS are electron-deficient radicals that are highly reactive; as a result, they can cause oxidative damage to cellular components including the cell membrane, DNA, and proteins. When such oxidative damage persists, intracellular signaling pathways are activated, ultimately causing chronic systemic inflammatory responses by exacerbating various pathological conditions, including cardiovascular disease and cancer [1,2].

An inflammatory response refers to the mechanism whereby immune cells inside the body secrete various inflammatory mediators upon recognition of external physical or chemical stimuli or bacterial infection in an effort to restore or regenerate damaged tissues [3]. However, excessive or prolonged inflammatory responses may cause chronic inflammatory diseases, such as arthritis, asthma, multiple sclerosis, chronic enteritis, and psoriasis [4]. In such inflammatory responses, macrophages are known to play a role in host defense during the early stage of an infection by producing inflammatory mediators, such as nitric oxide (NO), interleukin-1β (IL-1β), interleukin-6 (IL-6), and tumor necrosis factor-α (TNF-α) [5].

Many factors are known to be involved in regulating the inflammatory response inside the body; the nuclear transcription factors NF-κB and Nrf-2 are known to play an important role in the activation and inhibition of inflammatory responses [6,7]. When excessive levels of ROS are generated in the body, various signaling pathways are activated, including the activation of the NF-κB pathway, which causes the nuclear translocation of the NF-κB protein, which then promotes the expression of iNOS, COX-2, and inflammatory cytokines to accelerate the inflammatory response [8]. Nrf-2 is a nuclear transcription factor known to regulate the expression of phase 2 detoxification enzymes such as HO-1 and NQO-1, while the Nrf-2-mediated upregulation of phase 2 detoxification enzymes and anti-oxidative enzymes are known to eliminate ROS and inhibit inflammatory responses [9]. Recent studies on the anti-inflammatory effects of various polyphenol compounds in plants have reported that these compounds activate Nrf-2, thereby increasing the expression of phase 2 detoxification and antioxidative enzymes; thus, various physiological functions, such as antioxidative and anti-inflammatory actions, are performed [10].

*Calystegia soldanella* (L.) roem. et Schult, a perennial vine belonging to the family Convolvulaceae, is a halophyte that inhabits coastal sand dunes and even thrives in environments with high salinity [11]. For a long time, *C. soldanella* has been reported to exert antipyretic, disinfecting, and diuretic effects, as well as various other physiological activities, including antioxidative, anti-inflammatory, antiviral, and antifungal activities; it has also been known to inhibit the expression of protein tyrosine phosphate 1 B [12,13,14,15]. Compounds isolated from *C. soldanella* include resin glycoside, anthocyanins, caffeic acid, and coumaric acid [16,17]. While various physiological activities of *C. soldanella* have been reported, as described above, the Nrf-2 activation-mediated anti-inflammatory effects of *C. soldanella* extract and the mechanisms underlying these effects have not yet been reported. Thus, the research team of this study investigated the anti-inflammatory effects of *C. soldanella* extract and organic solvent fractions in Raw 264.7 mouse macrophages and the mechanisms underlying these effects to demonstrate the potential of extracts and organic solvent fractions from this plant to serve as a novel anti-inflammatory agent.

## 2. Materials and Methods

### 2.1. Materials

Antioxidant activity and NO concentration measuring reagents such as 2,2-Diphenyl-1-picrylhydrazyl (DPPH), 2,2′-azobis(2-methylpropionamidine) dihydrochloride (AAPH), 2,2′-Azino-bis(3-ethylbenzothiazoline-6-sulfonic acid) diammonium salt (ABTS), Trolox, Folin–Ciocalteu reagent, 2,4,6-Tris(2-pyridyl)-s-triazine (TPTZ), gallic acid, quercetin, trichloroacetic acid, aluminum chloride (AlCl_3_), thiazolyl blue tetrazolium bromide (MTT), and lipopolysaccharide (LPS) were purchased from Sigma-Aldrich (St. Louis, MO, USA). The mouse macrophage cell line, Raw 264.7, were obtained from the American Type Culture Collection (Manassas, VA, USA). Dulbecco’s modified Eagle’s medium (DMEM), fetal bovine serum, and antibiotics used Raw 264.7 cell culture were purchased from Wellgene (Daegu, Korea). All antibodies used in Western blotting were obtained from Santa Cruz Biotechnology (Santa Cruz, CA, USA).

### 2.2. Preparation of Extracts and Fractions

The dried powder of *C. soldanella* was extracted by reflux three times with 10× excess by weight of ethanol and distilled water for 12 h. After drying by evaporation of ethanol and distilled water in a vacuum rotary evaporator, the extract was made into an aqueous suspension and fractionated into the n-hexane fraction, dichloromethane fraction, ethyl acetate fraction, n-butanol fraction, and aqueous fraction three times. The five fractions were obtained after removal of the solvents.

### 2.3. Determination of Total Phenol and Flavonoid Content

The total phenolic compounds were quantified using a protocol similar to that described by Eom et al. [18]. Briefly, 0.1 mL aliquots of sample solutions were mixed with 3.5 mL of distilled water and 0.5 mL of 50% phenol reagent. The mixtures were then allowed to react for 2 h, after which 0.5 mL of 20% Na_2_CO_3_ was added. The mixture was then incubated in the dark for 1 h, and the absorbance was recorded at a wavelength of 720 nm using a Synergy HTX multi-mode microplate reader (Biotek, Winooski, VT, USA). The total phenolic content was expressed as gallic acid equivalents (mM GAE/g) dry sample and all determinations were carried out in triplicate.

Total flavonoid content was determination by the method of Pekal et al., with slight modifications [19]. Briefly, 0.5 mL of each sample solution was mixed with 0.1 mL of 10% (w/v) AlCl_3_ and 0.1 mL of 1.0 M potassium acetate. Then, 1.5 mL of ethanol and 2.8 mL of distilled water were added and mixed. The mixture was then incubated in the dark for 1 h, and the absorbance was recorded at a wavelength of 415 nm using a Synergy HTX multi-mode microplate reader. The total flavonoid content was expressed as quercetin equivalents (mM QE/g) dry sample and all determinations were carried out in triplicate.

### 2.4. TEAC Assay

The Trolox equivalent antioxidant capacity (TEAC) assay is based on the reaction of ABTS radical and was carried out according to the method of Zulueta et al., with minor modifications [20]. An ABTS radical working solution was prepared daily by diluting the ABTS radical stock solution with distilled water to obtain an absorbance of 0.07 ± 0.02 at a wavelength of 734 nm. Briefly, 0.1 mL of sample solution was mixed with 2.0 mL of ABTS radical working solution. The mixture was incubated in the dark for 5 min and the absorbance was measured using a Synergy HTX multi-mode microplate reader. The sample extract activity was expressed as mM Trolox equivalent (TE)/g dry sample and all determinations were carried out in triplicate.

### 2.5. ORAC Assay

The oxygen radical absorbance capacity (ORAC) assay measures antioxidant inhibition of peroxyl radical-induced oxidation and reflects radical chain breaking antioxidant activity by H atom transfer. This assay is based on the scavenging of peroxyl radicals generated by AAPH, preventing degradation of the fluorescein and consequently preventing loss of fluorescence. In this study, the method of Zulueta et al. [20] was employed. For sample dilution and reagent preparation, 75 mM phosphate buffer (pH 7.4) was used. Briefly, 50 μL of sample extract and 150 μL of 75 nM fluorescein solution were added to the wells of 96-well black-bottomed microplates and preincubated for 10 min at 37 °C. The reaction was initiated by the addition of 25 μL of 120 mM AAPH solution and the change in fluorescence was monitored with a Synergy HTX multi-mode microplate reader with excitation and emission wavelengths of 460 nm and 530 nm, respectively, for 60 min. The sample extract activity was expressed as mM of TE/g dry sample and all determinations were carried out in triplicate.

### 2.6. FRAP Assay

The ferric reducing antioxidant power (FRAP) was determined by the method described by Benzie et al., with slight modifications [21]. Briefly, 0.1 mL of sample extract was mixed with 3.0 mL of FRAP working reagent prepared fresh daily. FRAP working reagent consisted of 10 volumes of 300 mM acetate buffer (pH 3.6) and 10 volumes of 20 mM FeCl_3_. One volume of 10 mM TPTZ in 40 mM HCl was also added and the final mixture was incubated at 37 °C in the dark for 30 min. The absorbance at 593 nm was measured after 30 min. The activities were expressed as mM of FeSO_4_/g dry sample and all determinations were carried out in triplicate.

### 2.7. Cell Culture and Cell Viability Assays

Raw 264.7 cells were cultured in DME supplemented with 10% fetal bovine serum, antibiotics at 37 °C CO_2_ incubator. The medium was changed every other day. Cell viability was measured by MTT assay, which is based on the conversion of MTT to formazan crystals by mitochondrial dehydrogenases. Cells were cultured in 96-well plates (1.0 × 10^4^ cells/well) with serum-free medium and treated with different concentrations of sample for 24 h. The extract of *C. soldanella* and its solvent fractions were dissolved in 10% DMSO. The final concentration of DMSO in the culture medium never exceeded 0.1%. A 100 μL aliquot of MTT dye solution was added to each well. After 2 h of incubation, 200 μL of DMSO was added to dissolve the formazan crystals and the absorbance at 540 nm was read using a Synergy HTX multi-mode microplate reader.

### 2.8. Quantification of NO Production

Raw 264.7 cells were cultured in 96-well plates in DMEM medium without phenol red and pretreated for 1 h followed by treatment with test materials. Cellular NO production was induced by adding LPS to a final concentration of 1 μg/mL, followed by incubation for 24 h. After incubation, 100 μL of conditioned medium containing nitrite was mixed with the same volume of Griess reagent and incubated for 15 min. The absorbance of the mixture at 550 nm was measured with a Synergy HTX multi-mode microplate reader.

### 2.9. Cytokine Analysis

Cells were treated with different concentrations of test materials for 1 h, and IL-1β, IL-6, and TNF-α production were stimulated by addition of 1 μg/mL of LPS and incubation for a further 24 h. The supernatant was collected, and IL-1β, IL-6, and TNF-α production were quantified by sandwich immunoassays using Quantikine ELISA kits (R&D Systems, Minneapolis, MN, USA) according to the manufacturer’s instructions.

### 2.10. Total, Nuclear Protein and Cytosol Protein Isolation

Total protein was isolated using RIPA buffer as follow: Raw 264.7 cells were cultured in Dulbecco’s modified Eagle medium at a density of 1 × 10^5^ cells in 10-cm^2^ cell culture dishes and incubated for 24 h. The cells were treated with different concentrations of CsEF with 1 μg/mL of LPS for 24 h. The cells were lysed with RIPA buffer (Sigma-Aldrich, St. Louis, MO, USA) and supernatants with protease and phosphatase inhibitor cocktail were centrifuged at 2000× *g* for 10 min to remove insoluble materials. Nuclear and cytosolic proteins were separated using the NE-PER Nuclear protein extraction kit (Thermo Fisher Scientific, Rockford, IL, USA) according to the manufacturer’s instructions. The concentrations of protein in the supernatants were determined using a BCA protein assay kit (Thermo Fisher Scientific, Rockford, IL, USA).

### 2.11. Western Blot

The same amounts of cell lysates were analyzed by 10% sodium dodecyl sulfate polyacrylamide gel electrophoresis (SDS-PAGE) and the proteins were blotted onto nitrocellulose membranes and blocked with 3% bovine serum albumin in Tris-buffered saline containing 0.1% Tween 20 (TBS-T) for 1 h. Subsequently, the primary monoclonal antibodies were added to the TBS-T and incubated overnight. Proteins were detected using horseradish peroxidase-conjugated secondary antibody and enhanced using a chemiluminescence ECL assay kit (Bio-Rad, Hercules, CA, USA) according to the manufacturer’s instructions, and imaged on a GeneGnome 5 image analysis (Synoptics, Cambridge, UK). The basal levels of the proteins were normalized relative to the level of β-actin protein.

### 2.12. Real Time Quantitative Polymerase Chain Reaction

Raw 264.7 cells were cultured in 10 cm^2^ cell culture dishes and incubated for 24 h. The cells were treated with different concentrations of CsEF with 1 μg/mL of LPS for 24 h. Total RNA was extracted from treated cells using Trizol reagent (Thermo Fisher Scientific, Rockford, IL, USA). Furthermore, 1 μg total RNA was converted to cDNA using ReverTra Ace reverse transcriptase (Toyobo, Osaka, Japan) according to the manufacturer’s instructions and the cDNA samples were stored at −80 °C. Target gene amplification was performed in 20 μL reaction using SYBR Green Realtime PCR master mix (Toyobo, Osaka, Japan) and Step one plus real-time PCR system (Thermo Fisher Scientific, Rockford, IL, USA). All mRNA levels were normalized using glyceraldehyde-3-phosphate dehydrogenase (GAPDH) as an internal control. The primers used for amplification are shown in (Supplement Material Table S1).

### 2.13. UPLC Q-TOF Mass Analysis

The phytochemicals in the CsEF were analyzed using UPLC-quadruple time of flight (Q-TOF) mass spectrophotometry. The UPLC system (Waters infinity 1260 series, Germany), with an incorporated photodiode array detector (DAD) and Impact II Q-TOF mass spectrometer (Bruker Daltonik GmbH, Germany), was equipped with an ESI source that operated on the negative ion mode. A reverse phase Kintex core-shell C-18 column (100 × 2.1 mm, 1.7 μm, Phenomenex) was used at a flow rate of 0.5 mL/min. The mobile phase consisted of water containing 0.1% TFA (A) and 0.1% TFA containing acetonitrile (B) using the following gradient conditions 0–1 min, 10% B; 1–4 min, 10–20% B; 4–6 min, 20–25% B; 6–8 min, 25% B; 8–9 min, 25–30% B; 9–11 min, 30% B; 11–12 min, 30–50% B; 12–14 min, 50–60% B; 14–15 min, 60–80% B; and 15–17 min, 80% B. The injection volume was 2 μL. Mass spectra in positive-ion or negative-ion mode were recorded within 20 min. The UPLC profiles of the extracts were measured using a DAD. The analyses were conducted in the negative ion mode in a mass range from *m*/*z* 50 to 1000. The ESI source parameters were: capillary voltage, 4.5 KV; nebulizing gas pressure, 1.5 Bar; drying gas temperature, 200.0 °C, drying gas flow, 9.0 L/min; Funnel 1RF 250.0 Vpp; transfer time, 50.0 μs; and pre-pulse storage, 2.0 μs. The MS data were analyzed using Data Analysis 4.2 software (Bruker Daltonics, Bremen, Germany).

### 2.14. Statistical Analysis

Each experiment was performed at least three times and results are presented as the mean ± SD (standard deviation). Statistical comparisons of the mean values were performed using one-way ANOVA followed by Duncan’s multiple range test using Minitab 17 software (Minitab Inc., IL, State College, PA, USA). Differences were considered significant at *p* < 0.05.

## 3. Results

### 3.1. Polyphenol and Flavonoid Contents in C. Soldanella Ethanol Extract and Organic Solvent Fractions

Polyphenols are aromatic compounds that contain two or more phenolic hydroxyl groups. These can be classified as phenolic acids (e.g., caffeic acid and chlorogenic acid) and flavonoids (e.g., kaempferol and catechin). Various physiological functions of plant extracts, such as antioxidative and anti-inflammatory activities, have been reported to originate from compounds contained in the extract. Total polyphenol and flavonoid contents were measured, as shown in Table 1. Analysis of total polyphenol and flavonoid content in *C. soldanella* ethanol extract (CsEE) and fractions showed that the ethyl acetate fraction (CsEF) had the highest content of polyphenols, followed by the butanol fraction (CsBF) and CsEE, whereas the dichloromethane fraction (CsDF), hexane fraction (CsHF), and aqueous fraction (CsAF) had the lowest polyphenol contents. CsEF had the highest flavonoid content, followed by CsBF, CsEE, CsDF, CsHF, and CsBF.

### 3.2. Antioxidant Activity of C. soldanella Extract and Fractions

To measure the antioxidative activity of in *C. soldanella* ethanol extract and fractions, its electron- and hydrogen-donating abilities were measured. The electron-donating ability was measured using the TEAC and FRAP assays, while the hydrogen-donating ability was measured using the ORAC assay. Free radicals, including ROS, are unstable compounds with either electron, hydrogen atom, or both, deficiencies that attempt to become stable by accepting either an electron, hydrogen atom, or both. Accordingly, compounds with either high electron-, hydrogen atom, or both, -donating abilities are known to have high antioxidative activity. With respect to the TEAC and FRAP activities, which indicate the electron-donating ability (Table 2), CsEF showed the highest electron-donating ability (TEAC: 2281.09 TE mM/g, FRAP: 3193.96 Fe^2+^ mM/g), followed by CsDF, CsBF, CsDF, CsEE, CsHF, and CsAF (in decreasing order). With regard to the ORAC test, which indicates the hydrogen-donating ability, similar to the case for the electron-donating ability, CsEF showed the highest hydrogen-donating ability (40.15 mM/g), followed by CsDF, CsBF, CsDF, CsEE, CsHF, and CsAF (in decreasing order).

### 3.3. Anti-Inflammation Activity of C. soldanella Extracts and Fractions

**Figure 1:**
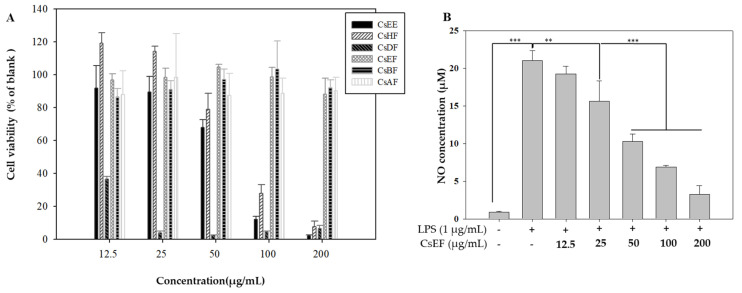
Cell viabilities of *C. soldanella* ethanol extracts and fractions in Raw 264.7 cell (**A**). Raw 264.7 cells were treated with various concentrations (12.5, 25, 50, 100, 200 μg/mL) of *C. soldanella* ethyl acetate fractions for 24 h. Cell viability was measured by MTT assay. (**B**) Nitric oxide production of CsEF in Raw 264.7 cells. Raw 264.7 cells were pre-incubated with 12.5, 25, and 200 μg/mL of extracts and fractions for 1 h and then treated with 1 μg/mL of LPS for 24 h. The NO production was measured by the Griess reagent system. Data are represented as means ± SE. The different superscripts are significantly different at *p* < 0.05. * Statistical significance of the difference between LPS and LPS + sample treatment groups: ** *p* < 0.01, *** *p* < 0.001.

To assess the anti-inflammatory activity of *C. soldanella* extracts and fractions, we measured their cytotoxicity in mouse leukemic monocyte macrophage cells (Raw 264.7). Raw 264.7 cells were treated with 12.5–200 μg/mL of *C. soldanella* extracts and fractions, and cell viability was measured after 24 h using the MTT assay. Cell viability of *C. soldanella* extracts and fractions are summarized in Figure 1A. The CsEF, CsBF, and CsAF did not show cytotoxicity within the indicated concentration range. The CsDF showed the highest cytotoxicity, decreasing cell survival to 36.63%, 3.90%, 2.63%, 4.41% and 6.59% at 12.5, 25, 50, 100 and 200 μg/mL, respectively. The CsEE and CsHF were not cytotoxic at a concentration of 12.5 and 25 μg/mL, but cell viability decreased at concentrations of 50–200 μg/mL. In the case of CsEF, which showed the greatest antioxidant activity, it did not show cytotoxicity at any concentration.

We assessed the inhibition of NO production induced by LPS at non-cytotoxic concentrations (12.5 and 100 µg/mL) for CsEF (Figure 1B). Based on the results of the NO inhibition assay, we used the CsEF for further cell-based experiments.

### 3.4. CsEF Inhibits the LPS-Mediated Overexpression of Inflammatory Cytokines in Raw 264.7 Cells

**Figure 2:**
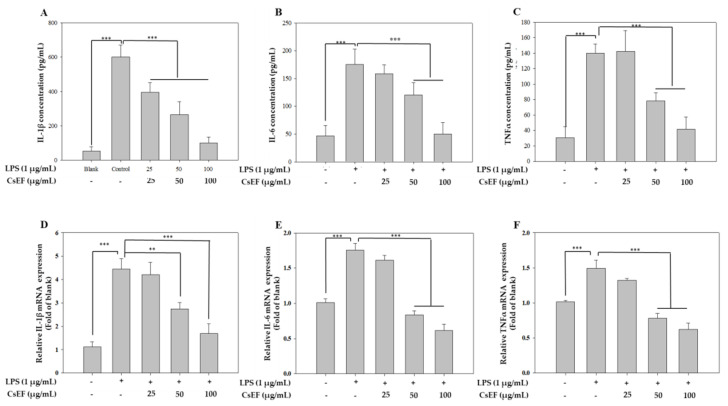
Inhibition of LPS induced inflammatory cytokine production in the CsEF in Raw 264.7 cell. Raw 264.7 cells were preincubated with 12.5 or 200 μg/mL of CsEF for 1 h and then treated with 1 μg/mL of LPS for 24 h. The IL-1β (**A**), IL-6 (**B**), and TNF-α (**C**) production was measured by ELISA, as described in Materials and Methods. Data are represented as means ± SEMs. * Statistical significance of the difference between LPS and LPS + sample treatment groups: ** *p* < 0.01, *** *p* < 0.001. The IL-1β (**D**), IL-6 (**E**), and TNF-α (**F**) mRNA transcription were analyzed using RTCsEF contain phenolic compounds using the indicated primer. GAPDH served as the internal standard gene. Data are represented as means ± SEMs. * Statistical significance of the difference between LPS and LPS + sample treatment groups: ** *p* < 0.01, *** *p* < 0.001.

Cytokines play a pivotal role in inflammatory responses by directly affecting the proliferation and activity of immune cells. Figure 2A–C shows the inhibition of IL-1β, IL-6, TNF-α secretion following CsEF treatment using enzyme-linked immunosorbent assay (ELISA). The secretion of pro-inflammatory cytokines by Raw 264.7 cells was sharply increased following LPS stimulation and decreased after treatment with EAF in a concentration-dependent manner. IL-1β, IL-6, and TNF-α were suppressed by 28%, 65%, and 68%, respectively, when treated with 50 μg/mL CsEF. In addition, RT-PCR was performed to determine whether CsEF-induced inhibition of IL-1b, IL-6, and TNFα production was due to the inhibition of the expression of these genes (Figure 2D–F). The results showed that the expression of these genes was inhibited by treatment with CsEF. Accordingly, it was determined that the CsEF-induced inhibition of pro-inflammatory mediators was due to the inhibition of the transcription of these genes and the expression of the corresponding proteins.

### 3.5. CsEF Inhibits the LPS-Mediated Overexpression of iNOS, COX-2 in Raw 264.7 Cells

**Figure 3:**
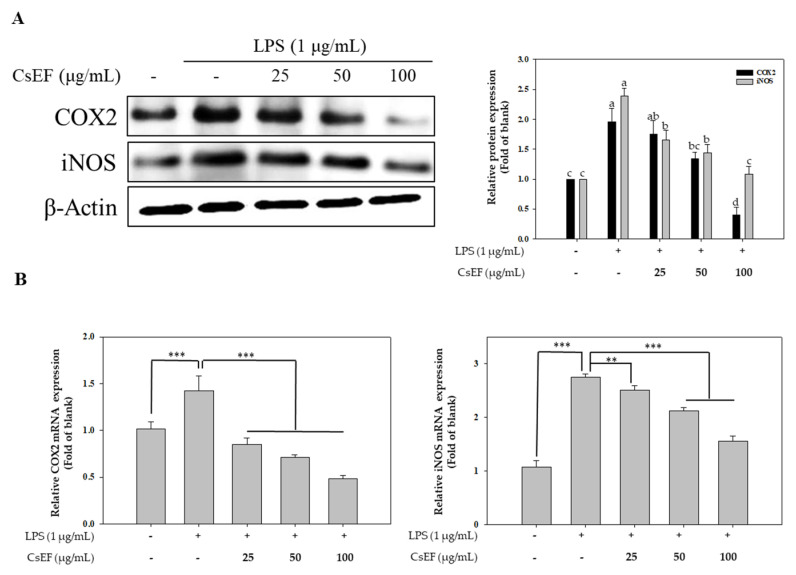
Effect of *CsEF* on COX2 and iNOS protein expression analysis by Western blotting (**A**) and mRNA translation analysis by RT-PCR (**B**) in LPS treated Raw 264.7 macrophage. Raw 264.7 cells were pre-incubated with 25–100 μg/mL of CsEF for 1 h and then treated with 1 μg/mL of LPS for 24 h. (**A**) Total protein extracts were analyzed using SDS-PAGE, followed by immunoblotting using the indicated antibodies. β-actin served as the internal cytosolic fractions. ^a–d^ Means with different superscripts in the same column are significantly different at *p* < 0.05. (**B**) The COX2, and iNOS mRNA transcription were analyzed using RT q-PCR using the indicated primer. GAPDH served as the internal standard gene. Data are represented as means ± SEMs. * Statistical significance of the difference between LPS and LPS + sample treatment groups: ** *p* < 0.01, *** *p* < 0.001.

Western blotting and RT-PCR experiments were performed to determine whether the CsEF-induced inhibition of NO and PGE2 production was due to the altered expression of NOS-2 and COX-2. Treating Raw 264.7 cells with LPS resulted in increased NOS-2 and COX-2 protein expression (Figure 3A) and mRNA transcription (Figure 3B). However, subsequent treatment with CsEF effectively inhibited the LPS-mediated increase in NOS-2 and COX-2 protein expression and mRNA transcription.

### 3.6. Effects of CsEF on NF-κB and MAPK Pathway Activation in LPS-Stimulated Raw 264.7 Cells

**Figure 4:**
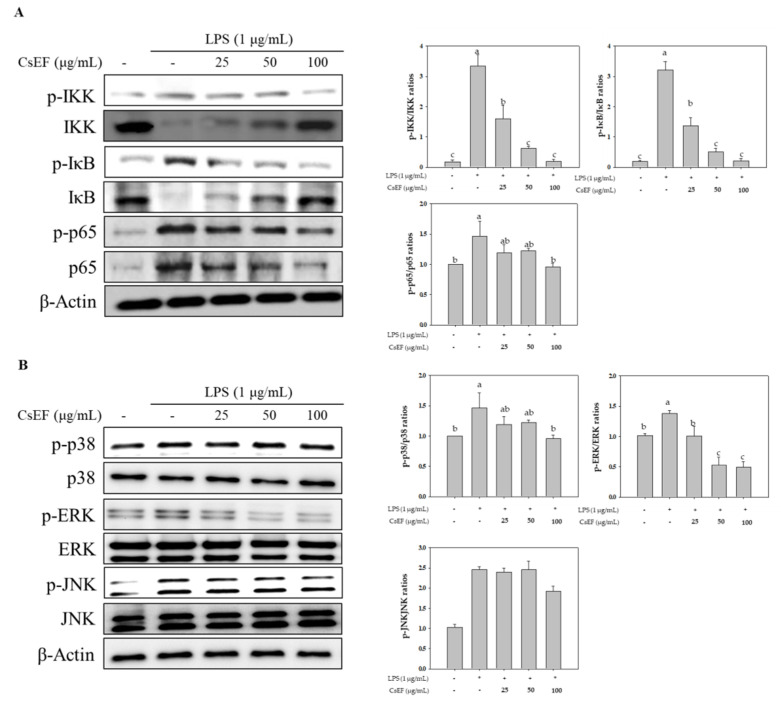
Effect of CsEF inhibits LPS-induced of NF-κB (**A**) and MAPK (**B**) pathway in Raw 264.7 cells. Raw 264.7 cells were pre-incubated with 25–100 μg/mL of CsEF for 1 h and then treated with 1 μg/mL of LPS for 24 h. Total protein extracts were analyzed using SDS-PAGE, followed by immunoblotting using the indicated antibodies. β-actin served as the internal cytosolic fractions. ^a–c^ Means with different superscripts in the same column are significantly different at *p* < 0.05.

The study also investigated whether the anti-inflammatory activity of CsEF was due to suppression of the NF-κB pathway. IKb-α, which is a protein that inhibits NF-κB expression, induces the nuclear translocation of NF-κB when it is phosphorylated upon LPS treatment. However, the phosphorylation of IkB-α was inhibited by CsEF treatment. In addition, the activity of IKK, a kinase that induces the phosphorylation of IκB-α, was also inhibited by CsEF treatment. Furthermore, NF-κB is activated by the phosphorylation of the NF-κB p65 subunit, which results in its nuclear translocation to induce the expression of various pro-inflammatory markers. Analysis of the phosphorylation of the NF-κB p65 subunit confirmed that NF-κB p65 phosphorylation was inhibited by CsEF treatment (Figure 4A).

NF-κB is activated by the activation of the MAPK signaling pathway, which promotes the nuclear translocation of NF-κB protein to induce the expression of pro-inflammatory mediators. Accordingly, the effects of CsEF on the MAPK signaling pathway were investigated. The results showed that phosphorylation of ERK, JNK, and p38 increased as a result of LPS treatment, while the LPS-induced phosphorylation of ERK was inhibited by CsEF treatment. However, the inhibition of JNK and p38 phosphorylation was not observed (Figure 4B).

These findings indicate that the anti-inflammatory activity of CsEF appeared as a result of the reduced activation of the NF-κB pathway via the inhibition of the LPS-stimulated phosphorylation of ERK.

### 3.7. Effects of CsEF on the Nrf-2 and HO-1 Pathway Activation in Raw 264.7 Cells

**Figure 5:**
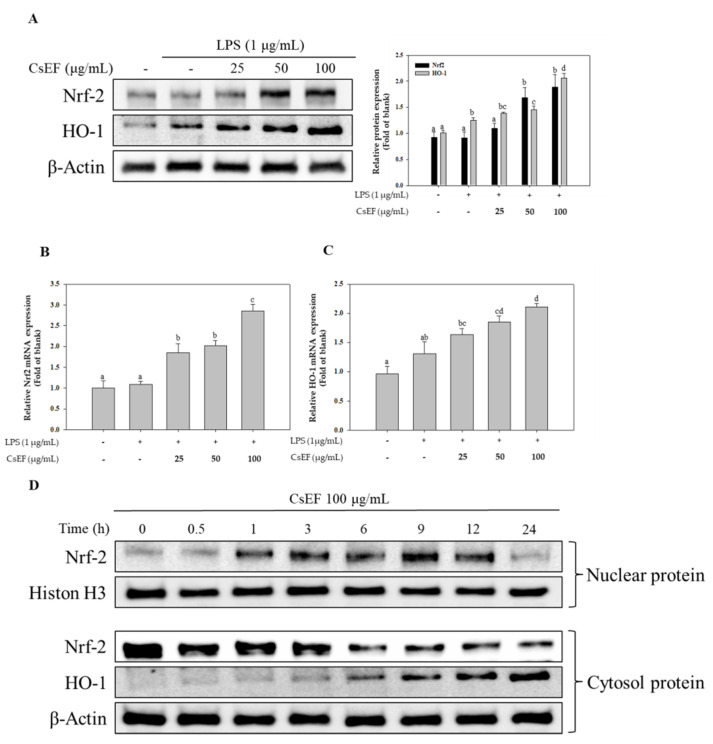
Effects of *CsEF* on the Nrf-2 and HO-1 protein expression and mRNA translation in Raw 264.7 cells. (**A**) Total protein extracts were analyzed using SDS-PAGE, followed by immunoblotting using the indicated antibodies. β-actin served as the internal cytosolic fractions. The Nrf-2 (**B**), and HO-1 (**C**) mRNA transcription were analyzed using RT q-PCR using the indicated primer. GAPDH served as the internal standard gene. Data are represented as means ± SEMs. ^a–d^ Means with different superscripts in the same column are significantly different at *p* < 0.05. (**D**) Nuclear and cytosolic fractions were analyzed using SDS-PAGE, followed by immunoblotting using several antibodies. Histon H3 and β-actin served as the internal controls for the nuclear and cytosolic fractions.

The study also investigated the association between the anti-inflammatory activity of CsEF and the Nrf-2 activation-mediated upregulation of HO-1. The results showed that CsEF treatment induced a concentration-dependent increase in Nrf-2 expression. Consequently, HO-1 expression also increased (Figure 5A,B). Moreover, because Nrf-2 is a nuclear transcription factor, its nuclear translocation was also investigated. The results showed that the nuclear translocation of Nrf-2 increased as a result of CsEF treatment (Figure 5A). These results indicate that CsEF regulates HO-1 expression by increasing the nuclear translocation of Nrf-2 (Figure 5B). Therefore, it was determined that increased HO-1 expression due to CsEF-induced Nrf-2 activation is associated with anti-inflammatory activity.

### 3.8. ESI-Q-TOF-MS Analysis of CsEF

According to previous studies, *C. soldanella* contains various polyphenol compounds, mainly flavonoids, flavonoid glycosides, and phenolic acid derivatives. To predict the substances that exhibit anti-inflammatory activity, UPLC-ESI-TOF-MS/MS was used to analyze the polyphenol compounds in CsEF. The UPLC UV chromatograms (280 nm) and total ion current chromatograms for CsEF are shown in the (Supplement Material Figures S1–S3). Table 3 shows the compounds analyzed based on molecular ion mass observed in the negative ion mode, fragment ion mass observed in MS/MS analysis, and MS data.

Q-TOF MS analysis results showed that the major compounds found in CsEF were the quercetin glycosides rutin, kaempferol, rutinoside, and quercetin. With respect to phenolic acids, the caffeic acid derivatives chlorogenic acid, dicaffeoylquinic acid, and 4-glycosyl-caffeoylquinic acid were identified. At a retention time of 2.1 min, a molecular ion peak of 609 (M-H)- was observed, and an *m*/*z* 301 (M-Rha-Glu-H)- group in which rhamnose and glucose were separated from (M-H)- appeared. Based on this, it was confirmed that rhamnose and glucose are combined in flavonoid glycosides. The MS/MS analysis of the peak corresponding to *m*/*z* 301 confirmed that the compound was quercetin, a flavonoid with a molecular weight of 302. Based on these results, it was confirmed that the compound with a retention time of 2.1 min was rutin, in which glucose and rhamnose were bound to quercetin.

At a retention time of 2.6 min, a molecular ion peak with an *m*/*z* value of 593.51 (M-H)- was observed.

Similar to rutin, an *m*/*z* 285.03 (M-glu-rha-H)- peak with reduced rhamnose and glucose in (M-H)- was observed. This was confirmed as a flavonoid glycoside with a molecular weight of 286, in which glucose and rhamnose are bound to a flavonoid. The *m/z* 285.03 peak for the flavonoid aglycone (molecular weight, 286) obtained after MS/MS analysis was actually kaempferol. Thus, it was confirmed that the kaempferol rutinoside was present. At a retention time of 3.0 min, a molecular ion peak (M-H)- with an *m/z* value of 447.09 was observed. The *m*/*z* value of 301 decreased by 146 in (M-H)^-^. This means that rhamnose is separated from (M-H)-. In addition, it was confirmed that the *m/z* value of 301 indicated the presence of quercetin, which has a molecular weight of 302, similar to the case for rutin; quercetin was identified based on this reasoning.

With respect to phenolic acid derivatives, peaks appeared at 1.5 and 1.6 min and MS analysis in negative ion mode generated the 353 (M-H)- peak, which was identified to be caffeoylquinic acid, with caffeic acid bonded to quinic acid based on same molecular ion. Moreover, the same 515.11 (M-H)- appeared at retention times of 2.8, 3.0, 3.2, and 3.6 min, and based on the *m/z* 353 molecular ion peak that appeared after the removal of a single molecule of caffeic acid, a dicaffeoylquinic acid derivative with two caffeic acid molecules bonded to quinic acid. Comparative analysis was performed against the results from previous studies to determine the substitution position of caffeic acid in dicaffeoylquinic acid [22].

At retention times of 3.2 and 3.6 min, fragment ion peaks corresponding to *m/z* 173 that appeared from the separation of two caffeic acid molecules were commonly observed. These findings confirmed that the structure had a substitution of caffeic acid at position 4 of quinic acid. However, a fragment ion peak corresponding to *m/z* 353 with the removal of one caffeic acid group appeared strongly at 3.2 min, but weakly at 3.6 min; accordingly, the peaks were predicted to be 1,4-dicaffeoylquinic acid and 3,4-dicaffeoylquinic acid, respectively.

At retention times of 2.8 and 3.2 min, fragment ion peaks corresponding to *m/z* 191 were commonly observed, which confirmed the presence of dicaffeoylquinic acid derivative with no acyl group on position 4 of quinic acid. Moreover, a fragment ion peak corresponding to *m/z* 353, with the separation of a single caffeic acid molecule, was observed weakly at 2.8 min, but strongly at 3.0 min. Accordingly, the peaks were predicted to be 1,5-dicaffeoylquinic acid and 1,3-dicaffeoylquinic acid, respectively.

## 4. Discussion

Polyphenol compounds found in various medicinal crops have recently been recognized as having the ability to inhibit oxidative stress-induced inflammatory responses and prevent various diseases caused by chronic inflammatory responses [23]. Accordingly, various studies have been conducted on the use of such compounds as natural medicinal ingredients. While there have been many studies using herbal medicinal ingredients, studies using various halophytes inhabiting coastlands are still lacking. With the recent knowledge of various halophytes having potent physiological functionalities, many studies have been conducted on the use of halophytes in the development of medicinal ingredients [24,25]. In particular, extensive research *Salicornia herbacea* has identified potent anti-oxidative, anti-inflammatory, anti-cancer, and antidiabetic effects [26]. However, studies on *C. soldanella* are lacking. Accordingly, this study investigated the anti-oxidative and anti-inflammatory activities of *C. soldanella* to gather data on its potential for the development of functional ingredients.

Free radicals, including ROS and reactive nitrogen species (RNS), are unstable substances that are missing a single electron, which attempt to become stable by accepting an electron or a hydrogen atom from another substance. Therefore, the excellent electron-, hydrogen, or both, -donating ability and high anti-oxidative activity of certain compounds could be explained by their ability to effectively stabilize various types of radicals and ROS [27]. Various polyphenol compounds have excellent anti-oxidative activity because they have phenolic hydroxyl groups that can donate an electron or hydrogen atom to a radical and remain stable due to resonance stabilization [28]. In this study, CsEF, which was rich in polyphenol compounds showed the highest anti-oxidative effect; this was determined to be the result of the high polyphenolic compound content in CsEF. Moreover, these findings were consistent with those of other studies reporting that halophyte plant extracts with high polyphenol contents showed excellent anti-oxidative activity [11,29].

The anti-oxidative activities of various plant extracts were proportional to the polyphenol compound contents in the extracts [30]. Another study reported that among extracts and solvent fractions of *Salicornia europaea*, another halophyte, the ethyl acetate fraction had the highest polyphenol compound content, and as a result, the ethyl acetate fraction showed superior anti-oxidative activity, compared to that of the other fractions [31].

Macrophages are immune cells that induce immune responses by secreting NO and cytokines in response to external stimuli or eliminating foreign substances by phagocytosis. When such a response persists, it can develop into chronic inflammation, which can cause various diseases [5]. In the present study, CsEF, with excellent anti-oxidative activity, exhibited anti-inflammatory effects by inhibiting NO, inflammatory cytokine, and PGE2 generation. NO is generated by nitric oxide synthase (NOS). NOS includes three components, eNOS, nNOS, and iNOS, of which iNOS is known to play an important role in the inflammatory response [32]. CsEF-induced inhibition of NO production was found to be the result of the CsEF-induced inhibition of iNOS mRNA and protein expression.

The NO generated inside cells reacts with ROS to generate RNS, which are known to cause oxidative damage to biomolecules and accelerate inflammation-induced cell damage by impairing mitochondrial functions [33,34]. Various studies have reported that substances with excellent anti-oxidative activity can inhibit NO generation by eliminating ROS [35]. In this study, CsEF, with excellent anti-oxidative activity, was predicted to inhibit NO generation by LPS-stimulated ROS inhibition.

PGE2 is a substance produced by the inflammatory response, which is synthesized from arachidonic acid present on the cell membrane, while COX-2 is an enzyme that plays an important role in this process [36,37]. Increased PGE2 generation by COX-2 is known to accelerate the inflammatory response by inducing the expression of various inflammatory cytokines, such as IL-1b and TNF-α [38]. Moreover, NO generated by iNOS plays an important role in COX-2 expression [39]. The results of this study showed that CsEF inhibited PGE2 generation in a concentration-dependent manner, which was due to a decrease in COX-2 expression.

Il-1β, IL-6, and TNF-α are typical inflammatory cytokines secreted by activated macrophages; they activate other immune cells or accelerate the activation of macrophages by autocrine or paracrine action [40]. Moreover, excessive secretion of inflammatory cytokines promotes apoptosis, which causes tissue damage [41]. In this result, CsEF was found to reduce the secretion of these cytokines and their mRNA expression in LPS-stimulated macrophages. In summary, CsEF, with excellent anti-oxidative activity, inhibited NO generation and the secretion of various cytokines, which resulted in anti-inflammatory activity. A previous study on the anti-inflammatory activity of extracts and fractions of *Salicornia europaea*, another type of halophyte, also reported similar results [31]. Therefore, these findings suggest that various polyphenol compounds in CsEF could be used as anti-inflammatory agents; the potent antioxidative activity of CsEF may be attributed to these compounds.

The nuclear transcription factor NF-κB is known to play an important role in regulating the expression of various pro-inflammatory mediators [42]. Accordingly, studies on the inhibition of NF-κB activation play an important role in the pharmacological mechanisms underlying the action of anti-inflammatory drugs [43]. Under various inflammatory stimuli, such as LPS, NF-κB activation induces the phosphorylation and degradation of IκB-α and the nuclear translocation of the NF-κB p65 protein. Translocated p65 binds to the NF-κB binding site, activating the transcription of pro-inflammatory mediators [44]. We confirmed that the phosphorylation of IkB-a and nuclear translocation of the NF-κB p65 protein were suppressed by CsEF treatment. Moreover, the activation of MAPK signaling pathways, such as the ERK1/2, p38, and JNK pathways, plays an important role in regulating the NF-κB pathway. Moreover, phosphorylation of MAPK by various external stimuli activates the MAPK signaling pathway [44]. Therefore, MAPK signaling pathway regulators could be used as potent anti-inflammatory agents in the development of anti-inflammatory drugs [43]. In this study, CsEF notably reduced ERK1/2 phosphorylation. However, it had no effect on the components of other MAPK signaling pathways, such as the JNK and p38 pathways. These findings indicated that CsEF selectively suppressed the ERK signaling pathway, among various MAPK signaling pathways, to inhibit the activation of the NF-κB pathway.

Heme oxygenase-1 (HO-1) is an enzyme that catalyzes the decomposition of heme into biliverdin, iron, and carbon monoxide. Biliverdin is known to exhibit potent antioxidant activity [7,9]. Moreover, recent studies have reported that various polyphenol compounds in medicinal plants increase HO-1 expression, which in turn, increases anti-oxidative activity and inhibits inflammatory responses. Overexpression of HO-1 prior to inflammatory stimulation has been reported to inhibit the expression of pro-inflammatory mediators, such as NO and IL-6 [45]. Moreover, severe inflammation was observed in the HO-1 knockout mouse model [46]. Such experimental evidence suggests that HO-1 could be a potential molecular target for the treatment of inflammation [47]. HO-1 expression is regulated by Nrf-2, a nuclear transcription factor that is activated by intracellular oxidative stress and undergoes nuclear translocation to induce the expression of phase 2 detoxifying enzymes, such as HO-1. The Nrf-2 activation-induced upregulation of phase 2 detoxification enzymes plays an important role in eliminating oxidative stress inside the body by neutralizing oxidative stress and toxins [10]. In this study, we confirmed increased Nrf-2 expression and nuclear translocation, and the subsequent increase in HO-1 expression in CsEF-treated Raw 264.7 cells. These findings indicate that the anti-inflammatory activity of CsEF had a significant effect on HO-1 expression through the intracellular activation of Nrf-2.

To identify the polyphenolic compounds that influence the antioxidative and anti-inflammatory activities of CsEF, we performed UPLC Q-TOF MS/MS to analyze the polyphenolic compounds. Three types of flavonoid glycosides and six types of phenolic acid derivatives were identified. The flavonoid glycosides identified were quercitrin, isoquercitrin and rutin, with glucose bonded to the quercetin backbone, and kaempferol-3-rutinoside, with glucose bonded to the kaempferol backbone. To the best of our knowledge, thus far, no studies have reported the contents of these compounds in *C. soldanella*; hence, this was researched for the first time by our research team in the present study. Rutin and quercetrin are glycosides with quercetin aglycon as the backbone; they are found in high concentrations in various citrus fruits and onions [48]. In particular, these compounds have various physiological functions and have been studied for their anti-inflammatory activities and the prevention of inflammatory diseases [49]. Similar to the case in our study, other studies have reported that rutin and quercetrin can prevent various inflammatory diseases by inhibiting NF-κB activity and activating the Nrf-2 pathway [50,51]. The phenolic acid derivatives identified included two types of caffeoylquinic acids, with caffeic acid bound to quinic acid, and four types of dicaffeoylquinic acids. While there have been studies on caffeic acid esters with a bond between caffeic acid and long-chain alcohols in *C. soldanella* [13]; studies on caffeoylquinic acid and dicaffeoylquinic acid have only been reported recently. Caffeoylquinic acid and dicaffeoylquinic acid, which are caffeic acid derivatives, are present at high levels in coffee; strong anti-oxidative and anti-inflammatory activities are known to be exhibited by caffeic acids [52,53]. Studies have reported that caffeoylquinic acid and dicaffeoylquinic acid activates Nrf-2, thereby promoting the expression of phase 2 detoxifying enzymes, which helps in preventing oxidative stress and inhibits NF-κB activation [54,55]. Moreover, various dicaffeoylquinic acids are known to exhibit potent anti-inflammatory activity in various inflammatory diseases, including colitis [56]. In our study, it was determined that the potent antioxidative and anti-inflammatory activities of CsEF were attributed to the high contents of these compounds in CsEF.

## 5. Conclusions

In the present study, which was regarding the development of a natural medicinal ingredient with anti-inflammatory effects, we examined *C. soldanella*, which has not been studied extensively thus far. The antioxidative and anti-inflammatory effects of *C. soldanella* extract and solvent fractions were investigated in mouse macrophages; the results showed that CsEF, which possessed a high content of polyphenol compounds, showed the strongest antioxidative and anti-inflammatory activities. Investigation of the mechanism underlying the anti-inflammatory activity of CsEF revealed that it inhibited NF-κB activation by inhibiting ERK phosphorylation in the MAPK signaling pathway. In addition, CsEF exhibited anti-inflammatory effects by increasing the expression of HO-1 through Nrf-2 activation. Our results confirmed that the antioxidative and anti-inflammatory effects of CsEF could be attributed to its constituent polyphenol compounds, such as rutin, quercetin, kaempferol-3-rutinoside, caffeoylquinic acid, and dicaffeoylquinic acid. Based on these findings, we propose that *C. soldanella* could be used as a natural medicinal ingredient with excellent antioxidative and anti-inflammatory effects.

## Figures and Tables

**Table 1:** Total phenolic and flavonoid contents of *Calystegia soldenella* extracts and fractions ^1^.

Samples ^2^	Total Phenols (mg GAE/g)	Total Flavonoids (mg QE/g)
CsEE	21.59 ± 2.30 ^d^	5.65 ± 0.60 ^c^
CsHF	21.05 ± 2.14 ^d^	3.11 ± 0.01 ^d^
CsDF	32.95 ± 1.66 ^c^	3.51 ± 0.10 ^d^
CsEF	173.71 ± 1.15 ^a^	55.92 ± 0.37 ^a^
CsBF	40.34 ± 2.17 ^b^	14.32 ± 0.11 ^b^
CsAF	11.77 ± 1.86 ^e^	0.94 ± 0.01 ^e^

^1^ Values are each expressed as a mean ± SD (n = 3). ^2^ EE: ethanol extracts. HF: n-hexane fractions. DF: dichloromethane fraction. EF: ethyl acetate fraction. BF: n-butanol fractions. AF: aqueous fraction. ^a–e^ Means with different superscripts in the same column are significantly different at *p* < 0.05.

**Table 2:** FRAP, TEAC, and ORAC values of *Calystegia soldenella* extracts and fractions ^1^.

Samples ^2^	FRAP (mM FeSO_4_/g)	TEAC (mM TE/g)	ORAC (mM TE/g)
CsEE	239.50 ± 18.5 ^c^	184.55 ± 7.02 ^c^	254.4 ± 33.4 ^bc^
CsHF	57.30 ± 19.02 ^d^	59.16 ± 7.61 ^d^	156.8 ± 36.7 ^c^
CsDF	357.24 ± 17.01 ^b^	310.54 ± 7.53 ^b^	574.5 ± 33.4 ^b^^c^
CsEF	3194 ± 58.2 ^a^	2281.1 ± 78.3 ^a^	5763.0 ± 868 ^a^
CsBF	377.14 ± 11.85 ^b^	298.84 ± 7.66 ^b^	1228.7 ± 52.6 ^b^
CsAF	42.53 ± 9.88 ^d^	13.85 ± 3.88 ^d^	38.48 ± 6.84 ^c^

^1^ Values are each expressed as a mean ± SD (n = 3). ^2^ EE: ethanol extracts. HF: n-hexane fractions. DF: dichloromethane fraction. EF: ethyl acetate fraction. BF: n-butanol fractions. AF: aqueous fraction. ^a–d^ Means with different superscripts in the same column are significantly different at *p* < 0.05.

**Table 3:** Identified phenolic components in *Calystegia soldenella* ethyl acetate fraction using UPLC Q-TOF analysis.

NO.	Proposed Compound	Molecular Formula	Rt (min)	(M-H)- Calc.	(M-H)- Exp.	MS Fragment
1	Quinic acid	C_7_H_12_O_6_	0.6	191.159	191.0599	173.04, 160.84, 127.01
2	Protocatechuic acid	C_7_H_6_O_4_	1.2	153.113	153.0190	109.02
3	Dihydrocaffeic acid	C_9_H_8_O_4_	1.3	181.0501	181.0501	163.03, 135.04
4	Caffeoylquinic acid	C_16_H_18_O_9_	1.5	353.303	353.0877	191.05, 173.04
5	Coumaroylquinic acid	C_16_H_18_O_8_	1.8	337.304	337.0924	191.05, 173.04
6	Feruloylquinic acid	C_17_H_20_O_9_	1.9	367.330	367.1027	191.05, 173.04
7	Rutin	C_27_H_30_O_16_	2.1	609.513	609.1465	300.02
8	Isoquercitrin	C_21_H_20_O_12_	2.4	463.371	463.0855	300.02
9	Kaempferol rutinoside	C_27_H_30_O_15_	2.6	593.514	593.1515	285.03
10	Dicaffeoyl quinic acid	C_25_H_24_O_12_	2.8	515.447	515.1191	353.08, 173.04
11	Dicaffeoyl quinic acid	C_25_H_24_O_12_	3.0	515.447	515.1199	447.09, 353.08, 191.05
12	Quercitrin	C_25_H_24_O_11_	3.1	447.372	447.0938	301.03
13	Dicaffeoyl quinic acid	C_25_H_24_O_12_	3.2	515.447	515.1189	447.09, 353.08, 161.05
14	Dicaffeoyl quinic acid	C_25_H_24_O_12_	3.6	515.447	515.1197	353.04, 173.04

## Data Availability

Data is available within the article and Supplementary Materials.

## Supplementary Materials

The following are available online at https://www.mdpi.com/article/10.3390/antiox10101639/s1, Figure S1: UV chromatogram and total ion chromatograms of CsEF contain phenolic compounds obtain by UPLC ESI-Q-TOF MS using negative ion mode, Figure S2: TOF MS/MS spectrum of flavonoid compounds contained in CsEF. Figure S3: TOF MS/MS spectrum of phenolic acid compounds contained in CsEF, Table S1: Primer sequences used for real time q-PCR.
