# Supplementary material for: Calystegia soldanella Extract Exerts Anti-Oxidative and Anti-Inflammatory Effects via the Regulation of the NF-κB/Nrf-2 Pathways in Mouse Macrophages
Source: Antioxidants (Basel). 2021 Oct 18;10(10):1639. doi: 10.3390/antiox10101639 (PMC8533082; doi:10.3390/antiox10101639)
Supplement: Supplementary file 1 [file antioxidants-10-01639-s001.zip › antioxidants-1395971-supplementary.pdf]

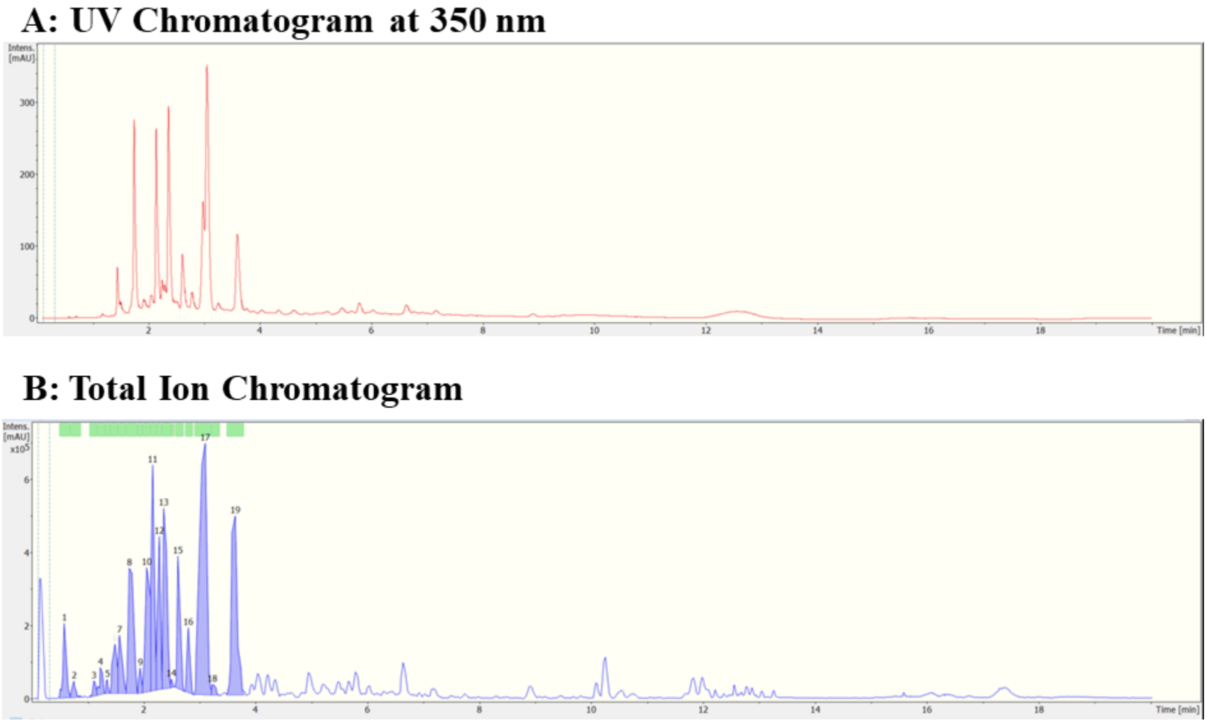

Figure S1. UV chromatogram and Total ion chromatograms of CsEF contain phenolic compounds obtain by UPLC-ESI-Q-TOF MS usning negative ion mode.

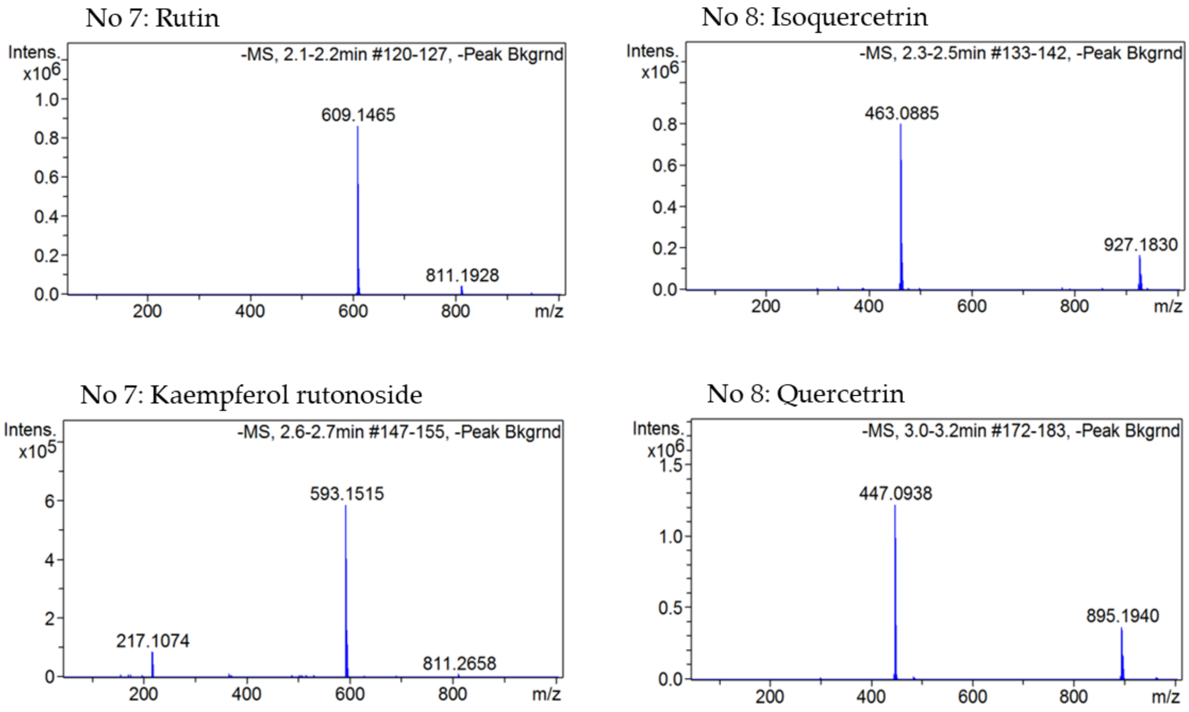

Figure S2. TOF-MS/MS spectrum of flavonoid compounds contained in CsEF.

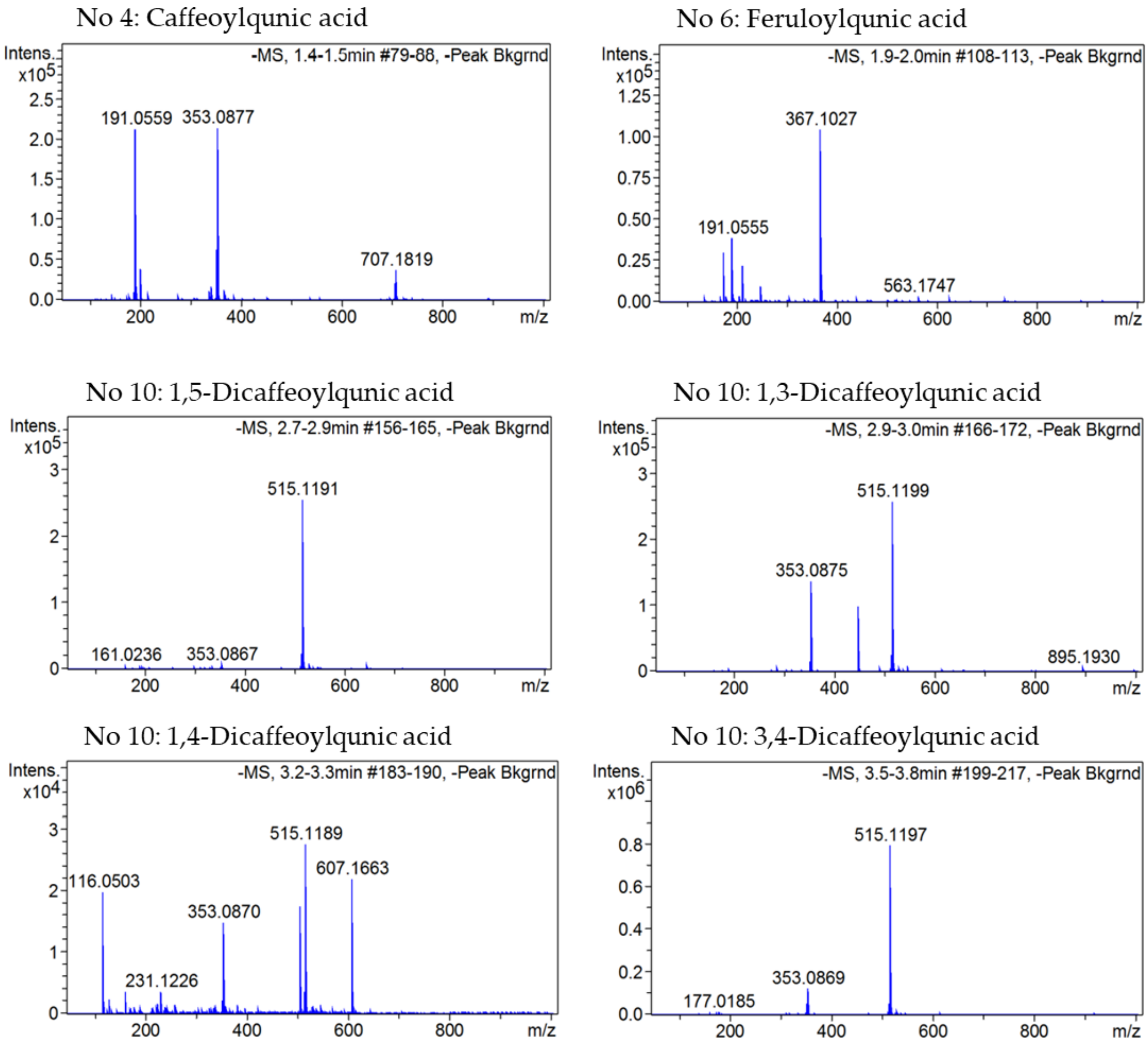

Figure S3. TOF-MS/MS spectrum of phenolic acid compounds contained in CsEF.

Table S1. Primer sequences used for real-time q-PCR.

Gene	Accession Number	Primer Sequences
IL-1β	NM_008361	F: 5'-TGACAGTGATGAGAATGACCTG-3'<br>R: 5'-CGAGATTTGAAGCTGGATGC-3'
IL-6	NM_031168	F: 5'-AACCGCTATGAAGTTCCTCTC-3'<br>R: 5'-TCCTCTGTGAAGTCTCCTCTC-3'
TNF-α	NM_013693	F: 5'-AGACCCTCACACTCAGATCA-3'<br>R: 5'-TCTTTGAGATCCATGCCGTTG-3'
COX2	NM_011198	F: 5'-CCAACCTCTCCTACTACACCA-3'<br>R: 5'-CTCCGTAGAAGAACCTTTTCC A-3'
iNOS	NM_010927	F: 5'-CTTGTTCAGCTACGCCTTCA-3'<br>R: 5'-TTGTCACCACCAGCAGTAG-3'
Nrf2	NM_010902	F: 5′-AGCAGGACATGGAGCAAGTT-3′<br>R: 5′-TTCTTTTTCCAGCGAGGAGA-3′
HO-1	NM_010442	F: 5′-CCCACCAAGTTCAAACAGCTC-3′<br>R: 5′-AGGAAGGCGGTCTTAGCCTC-3′
GAPDH	NM_008084	F: 5'-AATGGTGAAGGTCGGTGTG-3'<br>R: 5'-GTGGAGTCATACTGGAACATGTAG-3'
